# An enhanced version of the PHIRI infrastructure: improving the analytical services

**DOI:** 10.1093/eurpub/ckac129.468

**Published:** 2022-10-25

**Authors:** F Estupiñán

**Affiliations:** Data Sciences for Health Services and Policy, Institute for Health Sciences in Aragon, Zaragoza, Spain

## Abstract

The PHIRI federated approach has consisted of the development of four research queries (use cases) mobilising individual data from a number of data hubs (nodes in the federation). Methodologically speaking, use cases have required the creation of specific cohorts of patients, population subgroups or populations, and the identification of events of interest - over-time differences in health status and care healthcare utilisation before and during the pandemic. Technologically speaking, PHIRI infrastructure consists of a distributed end-to-end analytical pipeline containing the statistical analysis workflow, including data quality assessment at origin and the mathematical algorithms. Once datasets are prepared in each data hub, partners run the analyses and produce a research output (dashboards containing the research results and tables with aggregated data) that is shared for results compilation and comparative analysis. An enhanced version of the PHIRI infrastructure should allow more complex data distribution. The research questions covered so far are aiming inference on populations or providers, which implies a very simple distribution methodology, as described. However, when the research questions requires inference on the individuals (eg, quasi-experimental study on the effectiveness of a real-life intervention), when the inference requires a hierarchical approach (ie, part of the variance is at individual level and part at cluster level) or when, several rounds of training are needed (eg, validation of an artificial intelligence) the approach would require sharing coefficients, distances in n-dimensional spaces or models, and, some times various rounds of distribution. Finally, an enhanced version of the PHIRI infrastructure should generalise the current FAIR approach limited to the publication of the analytical pipeline in ZENODO, setting up the services and tools required for an improved version of the PHIRI open-science strategy.

